# Characterization of tumor-associated reactive astrocytes in gliomas by single-cell and bulk tumor sequencing

**DOI:** 10.3389/fneur.2023.1193844

**Published:** 2023-06-21

**Authors:** Chuan-bao Zhang, Zhi-liang Wang, Han-jie Liu, Zheng Wang, Wang Jia

**Affiliations:** ^1^Department of Neurosurgery, Beijing Tiantan Hospital, Capital Medical University, Beijing, China; ^2^The Chinese Glioma Genome Atlas (CGGA) Project, Beijing, China

**Keywords:** reactive astrocytes, glioma, molecular and genomic features, immunotherapy, sequencing

## Abstract

**Objective:**

Astrocytes constitute approximately 30% of cells in gliomas and play important roles in synapse construction and survival. Recently, JAK/STAT pathway activation associated with a new type of astrocyte was reported. However, the implications of these tumor-associated reactive astrocytes (TARAs) in glioma are not known.

**Methods:**

We comprehensively assessed TARAs in gliomas, both in single cells and at the bulk tumor level, by analyzing five independent datasets. First, we analyzed two single-cell RNA sequencing datasets of 35,563 cells from 23 patients to estimate the infiltration level of TARAs in gliomas. Second, we collected clinical information and genomic and transcriptomic data of 1,379 diffuse astrocytoma and glioblastoma samples from the Chinese Glioma Genome Atlas (CGGA) and The Cancer Genome Atlas datasets to evaluate the genomic, transcriptomic and clinical characteristics of TARA infiltration. Third, we downloaded expression profiles of recurrent glioblastoma samples from patients receiving PD-1 inhibitors to analyze the predictive value of TARAs for immune checkpoint inhibition.

**Results:**

Single-cell RNA sequencing data showed TARAs were abundant in the glioma micro-environment (15.7% in the CGGA dataset and 9.1% in the Gene Expression Omnibus GSE141383 dataset, respectively). Bulk tumor sequencing data showed that the extent of TARA infiltration was highly associated with major clinical and molecular features of astrocytic gliomas. Patients with more TARA infiltration were more likely to have *MUC16*, *FLG*, and *PICK3A* mutations, chromosome 9p21.3, 10q23.3, and 13q14.2 deletions and 7p11.2 amplification. Gene Ontology analysis revealed that the high level of astrocyte infiltration was characterized by immune and oncogenic pathways, such as the inflammatory response, positive regulation of the JAK–STAT cascade, positive regulation of NIK/NF-kappa B signaling and the tumor necrosis factor biosynthetic process. Patients with greater TARA infiltration showed inferior prognosis. Meanwhile, the extent of reactive astrocyte infiltration exhibited a predictive value for recurrent glioblastoma patients undergoing anti-PD-1 immune therapy.

**Conclusion:**

TARA infiltration might promote glioma tumor progression and can be used as a diagnostic, predictive and prognostic marker in gliomas. Prevention of TARA infiltration might be a new therapeutic strategy for glioma.

## Introduction

Glioma, arising from glial cells, are the most common malignant intracranial tumors in adults (1). The standard treatment strategy for glioma patients is surgical resection with adjuvant chemotherapy and/or radiotherapy (2, 3). However, the treatment often fails to prevent tumor recurrence or progression and the median survival for glioblastoma patients is less than 15 months (4).

Astrocytes account for nearly 30% of all cells in the human brain and are crucial in central nervous system (CNS) function (5), including formation and support of neuronal networks and construction of the brain–blood barrier (6, 7). In malignant tumors of the CNS, astrocytes are transformed into reactive astrocytes and are involved in many anti- and pro-tumor functions (8, 9). On the one hand, A1-specific reactive astrocytes participate in antigen presentation, complement activation and increased neurotoxicity. On the other hand, A2-specific reactive astrocytes release cytokines and molecules to exacerbate neuroinflammatory responses (10).

The tumor micro-environment has recently been recognized to be a pivotal element in tumorigenicity and Heiland et al. (11) identified a distinct reactive astrocyte subtype (tumor-associated reactive astrocytes, TARAs) marked by increased proliferation and JAK/STAT pathway activation. The characteristics of this type of reactive astrocyte in glioma are poorly understood; therefore, we characterized TARAs in gliomas by analyzing single-cell and bulk tumor sequencing data. The results may contribute to a full understanding of the glioma micro-environment and to the development of precision immune therapy.

## Materials and methods

### Single cell RNA sequencing data

Multi-sector biopsy-based single cell RNA sequencing data (12) of glioma samples were downloaded from the Chinese Glioma Genome Atlas (CGGA) database.^1^ These data were from 6,148 cells, involving 73 regions from 14 patients. We also downloaded an independent collection of single cell RNA sequencing data (13) from the Gene Expression Omnibus (GEO) database (GSE141383) for validation, which included data from 29,415 cells from nine glioma patients.

### Bulk tumor sequencing data and clinical information

We collected publicly available glioma gene expression data with full clinical information from two databases: the CGGA (China) (14) and The Cancer Genome Atlas (TCGA) (the United States^2^). To remove the impact of different tumor origins, patients diagnosed with oligodendroglioma, anaplastic oligodendroglioma, oligoastrocytomas and anaplastic oligoastrocytomas were excluded. We obtained 378 samples with clinical and molecular information from the CGGA database. A further 810 astrocytic glioma (grade II–III astrocytoma and glioblastoma) samples with clinical and molecular information (copy number, mutation, gene expression data) were downloaded from TCGA database. The details of patients are described in Table 1.

**Table 1:** The clinicopathological characteristics of glioma samples.

		CGGA (*n* = 378, %)	TCGA (*n* = 810, %)
Age	Median (range)	45 (13–76)	52 (10–89)
Gender	Male	227 (60)	479 (59)
	Female	151 (40)	331 (41)
KPS	Preoperative KPS ≥ 80	NA	446 (55)
	Preoperative KPS < 80	NA	121 (15)
	NA	NA	243 (30)
WHO grade	II	64 (17)	108 (13)
	III	65 (17)	152 (19)
	IV	249 (66)	550 (68)
Pathological type	A	64 (17)	108 (13)
	AA	65 (17)	152 (19)
	GBM	249 (66)	550 (68)
Molecular subtype	Neural	75 (20)	111 (14)
	Proneural	77 (20)	211 (26)
	Classical	69 (18)	178 (22)
	Mesenchymal	146 (39)	189 (23)
	NA	11 (3)	121 (15)
IDH status	Mutant	130 (34)	234 (29)
	Wild type	227 (60)	460 (57)
	NA	20 (6)	116 (14)
1p/19q status	Codel	23 (6)	35 (4)
	Intact	314 (83)	766 (95)
	NA	41 (11)	9 (1)
MGMT promoter methylation	Methylated	105 (28)	372 (46)
	Unmethylated	138 (37)	266 (33)
	NA	135 (35)	172 (21)

NA, not available.

Expression profiling of samples from 28 recurrent glioblastoma patients who received neoadjuvant anti-PD-1 immunotherapy were also downloaded from the GEO database (GSE121810) to explore the predictive value of TARA infiltration in immune checkpoint inhibition.

### Inference of tissue-infiltrating immune and stromal cell populations

To quantify the tissue-infiltrating immune and stromal cell populations in glioma samples, we applied the Microenvironment Cell Populations-counter (MCP-counter) algorithm, which allows for sensitive and specific discrimination of eight immune and two stromal cell populations, including CD3+ T cells, CD8+ T cells, cytotoxic lymphocytes, NK cells, B lymphocytes, cells originating from monocytes (monocytic lineage), myeloid dendritic cells, neutrophils, as well as endothelial cells and fibroblasts. MCP-counter is an objective method based on a methodological framework of transcriptome markers that outputs an abundance estimate per cell population that enables an inter-sample comparison. This procedure was performed using the MCPcounter R package (15).

### Evaluation of chromosome 1p/19q status

Chromosome 1p/19q status was inferred from gene expression on chromosome 1p and 19q from start to end using a Gaussian window smoothing function (16).

### Tumor-associated reactive astrocyte infiltration score

The infiltration scores of TARAs were calculated for 1,079 astrocytic glioma patients for whom mRNA expression data (RPKM values) were available using the reactive-astrocyte representative genes determined by Heiland et al. (11). Single sample gene set enrichment analysis (ssGSEA) was used to calculate the enrichment scores (11).

### Functional and pathway analysis

Gene annotation enrichment analysis of TARA-related genes was executed using the BP function in clusterProfiler R package (17). A *p* value <0.05 and a *q* value <0.05 were used as cutoff values. Differentially expressed genes between TARA low and TARA high groups were calculated using limma in R package (adjusted *p* value < 0.05). The gene set enrichment analysis (GSEA) results of RAS-low and RAS-high groups were visualized by enrichplot in R package. The corrplot package was used to explore the correlation between RAS and other relevant biological processes curated by Mariathasan et al. (18).

### Statistical analysis

The single-cell sequencing data were analyzed using a standard Seurat V4 pipeline (19). Associations between continuous variables were calculated using the Pearson correlation method. Differences between groups were estimated using unpaired Student’s *t* tests, one-way ANOVA, or chi-square tests. The Kaplan–Meier survival curve and log-rank test were applied using survminer R package to estimate the distribution of overall patient survival. The forestplot package was applied to depict the efficacy of RAS in subgroups. The timeROC package was used to generate time-dependent ROC curves to evaluate the 1 year predictive accuracy of RAS and PD-1 expression. Maftools package was used to depict the mutation landscape of glioma samples in TCGA dataset. All statistical analyses were executed using R software (version 3.6.2). A two-sided *p* value < 0.05 was considered statistically significant.

## Results

### TARAs are abundant in the glioma micro-environment

**Figure 1:**
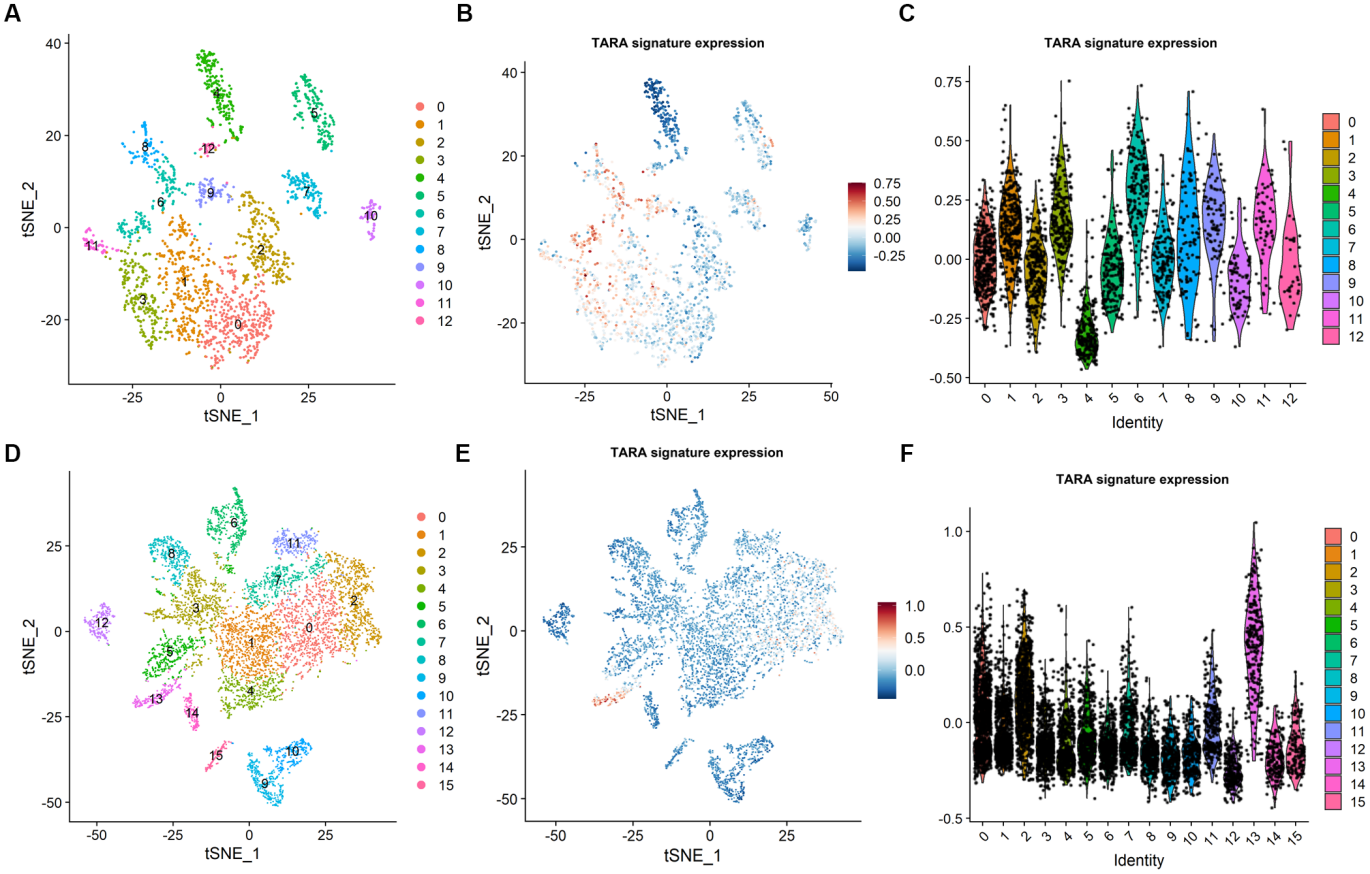
Tumor associated reactive astrocyte was abundant in glioma micro-environment. **(A)** Cell cluster in CGGA dataset. **(B)** TARA signature expression in CGGA dataset. **(C)** TARA signature expression in the clusters in CGGA dataset. **(D)** Cell clusters in GSE141383 data. **(E)** TARA signature expression in GSE141383 data. **(F)** TARA expression in the clusters in GSE141383 data.

To assess the possible infiltration of TARAs in gliomas, we first analyzed multi-sector biopsy-based single-cell RNA sequencing data of 6,148 cells. The type of the cells were first auto-annotated by SingleR package (Supplementary Figure S1) and the copy number of the cells were analyzed by inferCNV package (Supplementary Figure S2). The astrocytes showed glioma copy number features: 1p and 19q codeletion, 7 gain and 10 loss. To avoid potential contamination of malignant cells in the following analysis, the astrocytes were excluded. The remaining cells (*N* = 2,286) were well clustered into 13 clusters (Figure 1A, 0 ~ 12). To annotate TARAs among these cells, we used a previously published TARA signature, and found high expression of this signature in clusters 1, 3, 6, 8, 9, and 11 (Figures 1B,C). These cells accounted for approximately 43.4% (993/2,286) of non-malignant cells and 15.7% (993/6,148) of the total number of cells.

To validate these results, we analyzed GEO data of 29,415 cells from nine glioma patients (GSE141383). Similarly, the non-malignant cells (*N* = 7,243) were clustered into 16 clusters (0 ~ 15, Figure 1D). Clusters 0, 2, 7, 11, and 13 (2,685/7243, 37.1%; 9.1% (2,685/29,415) of total cells) showed high expression of the TARA signature (Figures 1E,F). These results indicated that TARAs abundantly infiltrated the glioma micro-environment.

### The clinicopathological characteristics of reactive astrocyte infiltration in glioma

**Figure 2:**
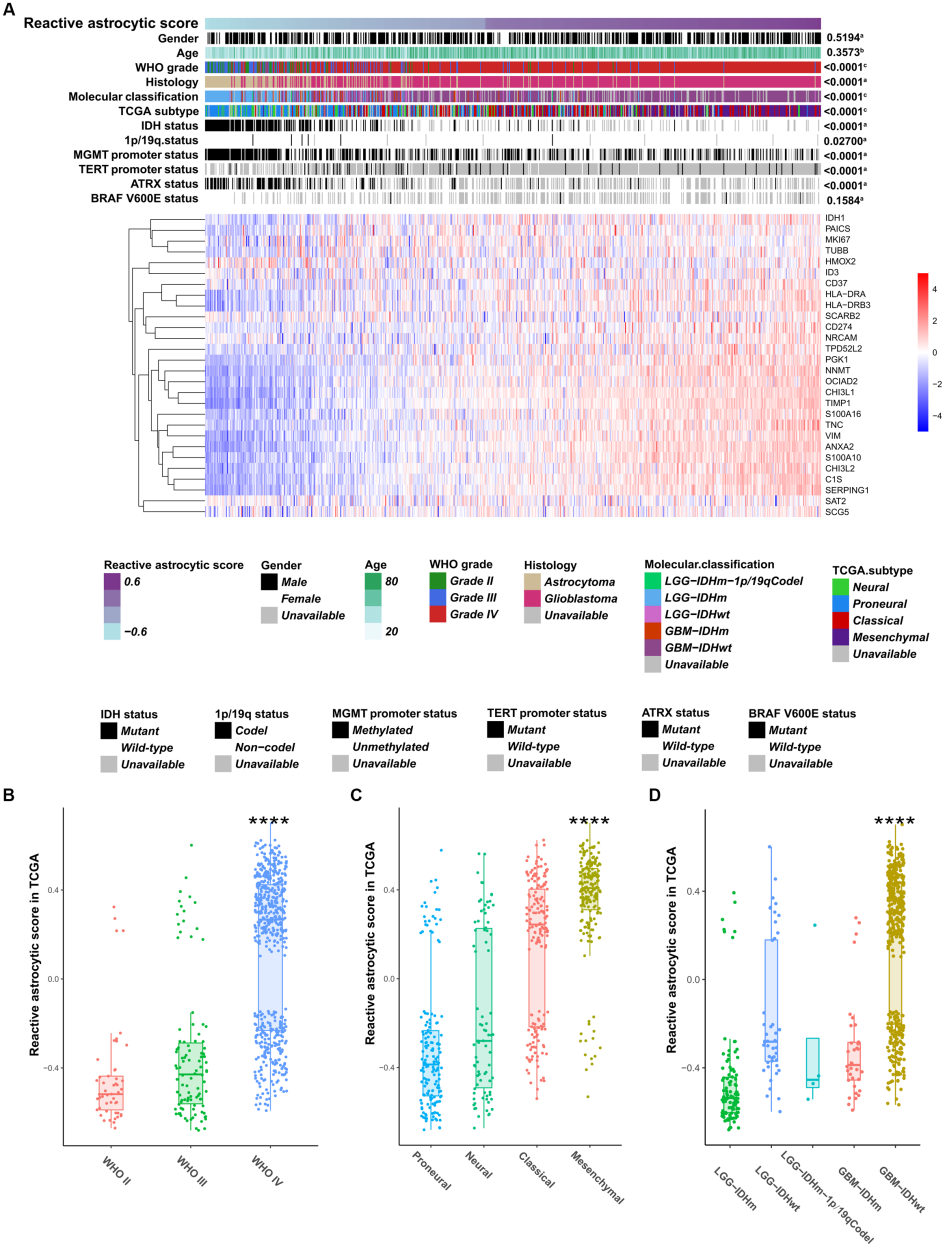
The clinical and molecular landscape of reactive astrocytic score in TCGA dataset. **(A)** The samples in heatmap were arranged in order of increasing reactive astrocytic score. The relationships between reactive astrocytic score and patients’ features were evaluated (a, the distribution of reactive astrocytic score was assessed using the student *t* test between two groups. b, The association between reactive astrocytic score and continuous variables was assessed using Pearson correlation tests. c, The distribution of glioma purity between several groups was assessed using one-way ANOVA). **(B)** The distribution of reactive astrocytic score among different WHO grades. **(C)** The distribution of reactive astrocytic score among different TCGA subtypes. **(D)** The distribution of reactive astrocytic score among different molecular classification. ****p < 0.0001.

To characterize and understand the biological and clinical features of reactive astrocyte infiltration, we quantified the relative abundance of TARAs in 1079 astrocytic glioma samples using the mRNA-based gene signature determined by Heiland et al. (11). The glioma samples were ranked according to increasing TARA infiltration (Figure 2A). Analysis of TCGA dataset revealed that WHO grade IV samples (Figure 2B), mesenchymal samples (Figure 2C) and *IDH* wild-type GBM samples (Figure 2D) were more likely to have more TARA infiltration. Meanwhile, correlations between the age at diagnosis and gender and reactive astrocytic score were not statistically significant (Figure 2A). In terms of well-known molecular markers, *IDH* mutation, 1p/19q codeletion, *MGMT* promotor methylation, wild-type *TERT* promoter, and *ATRX* mutation glioma samples correlated with lower infiltration of reactive astrocytes. Similar results were found in the CGGA dataset (Supplementary Figures S3A–D). In addition, recurrent glioma samples had a higher reactive astrocyte score than primary glioma samples. Moreover, when WHO grade (Supplementary Figure S3E), *IDH* status (Supplementary Figure S3F) and 1p/19q status (Supplementary Figure S3G) were added as sub-classifiers, the reactive astrocyte scores of recurrent glioma samples were significantly higher than those of primary glioma samples in the same subset. These results indicated that TARA infiltration was tightly associated with clinical and pathological features of glioma patients and might be a new diagnostic biomarker.

### Genomic characteristics of TARA infiltration

**Figure 3:**
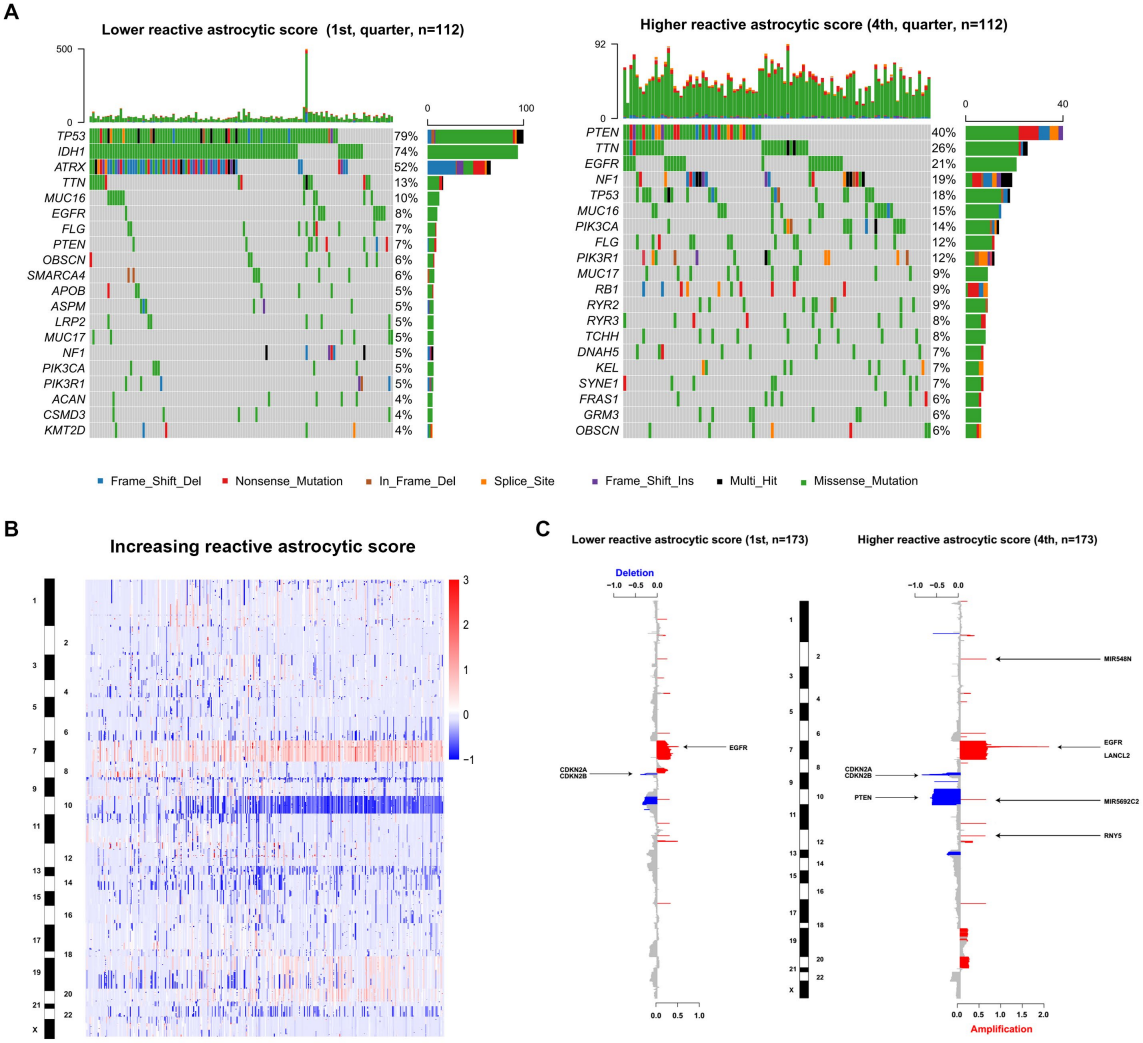
The genomic characteristics of reactive astrocytic score. **(A)** Differential somatic mutations were detected by comparing gliomas with low and high reactive astrocytic score. **(B)** The overall CNAs profile in order of increasing reactive astrocytic score. **(C)** The amplifications and deletions in gliomas with low and high reactive astrocytic score. Chromosomal locations of peaks of significantly recurring focal amplification (red) and deletions (blue) were presented.

To uncover genomic alterations associated with TARA infiltration, we analyzed somatic alterations and copy-number variations in TCGA dataset. Based on the increasing reactive astrocyte scores, we stratified TCGA glioma samples by defining tumors in the top 25% as the high TARA infiltration group (HRA) and the bottom 25% as the low TARA infiltration group (LRA), and we found mutated genes to occur significantly in each group. LRA gliomas had more mutations in *TP53*, *IDH1*, and *ATRX* (Figure 3A). Mutations in *PTEN*, *TTN*, *EGFR*, and *NF1* occurred more frequently in HRA samples (Figure 3A). We also observed a significantly different frequency of mutations in *MUC16*, *FLG*, and *PICK3A*.

Glioma is characterized by increased genomic instability with extensive copy number alterations (20, 21). We therefore assessed the copy number variations between the two groups. As shown in Figure 3B, the incidence of chromosome 7, 19, and 20 amplification, and chromosome 10 and 22 deletion were increased with increasing TARA infiltration. The HRA samples possessed more copy number variations, including deletions at 9p21.3 (*CDKN2A*/*CDKN2B*), 10q23.3 (*PTEN*/*ATAD1*) and 13q14.2 (*KPNA3*), and amplification of 7p11.2 (*EGFR*) (Figure 3C). Meanwhile, the recurrent copy number losses at 9p21.3 encompassing the *CDKN2A*/*CDKN2B* locus and at 10q26.3 encompassing the *MIR202* locus, and amplification at 7p11.2 encompassing the *EGFR* locus were the most common copy-number variations in LRA samples (Figure 3C). Globally, LRA samples were more stable and had significantly fewer somatic copy number alterations than HRA samples. These results indicated that TARAs tended to infiltrate gliomas with distinct genomic alterations.

### Functional annotation of TARA infiltration

**Figure 4:**
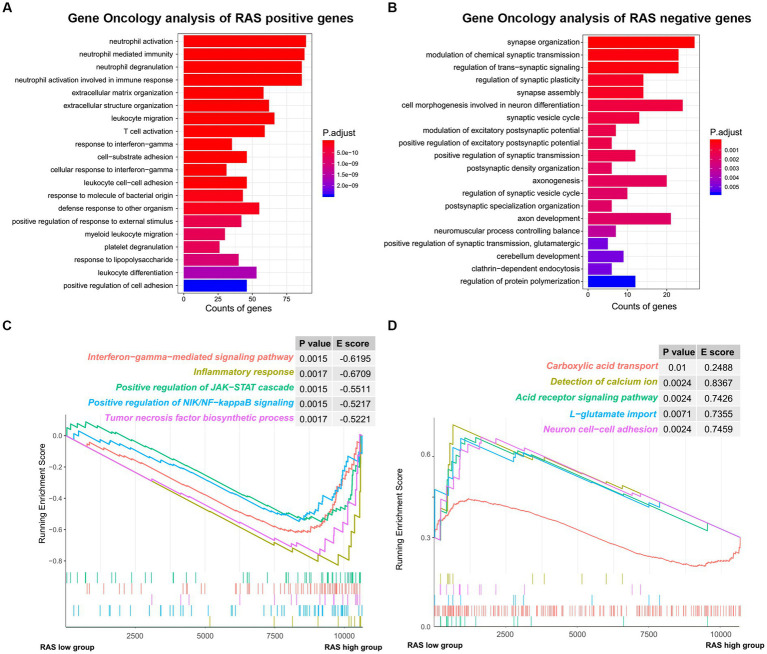
The functional analysis of reactive astrocytic score. **(A)** GO ontology analysis of RAS positive genes. **(B)** GO ontology analysis of RAS negative genes. **(C)** Enrichment plots showing the interferon gamma mediated signaling pathway, inflammatory response, positive regulation of JAK-STAT cascade, positive regulation of NIK/NF-kappaB signaling and tumor necrosis factor biosynthetic process gene sets in the reactive astrocytic score high group. **(D)** Enrichment plots showing the carboxylic acid transport, detection of calcium ion, acid receptor signaling pathway, L-glutamate import and neuron cell-cell adhesion in the reactive astrocytic score low group.

To reveal the functional characteristics of TARA infiltration, we identified the genes that were significantly positively (Pearson coefficient > 0.5) or negatively (Pearson coefficient < −0.5) correlated with reactive astrocyte score and annotated the gene sets using R package clusterProfiler. The top 20 significant biological processes are summarized in Figures 4A,B. Genes positively correlated with TARA scores were mainly involved in neutrophil activation, leukocyte migration, response to interferon gamma, T cell activation and other immune-related activations. The negatively correlated genes were enriched in synapse organization, modulation of chemical synaptic transmission, regulation of trans-synaptic signaling, cell morphogenesis involved in neuron differentiation, and other synaptic-signal-transduction-related biological processes.

Furthermore, GSEA of differentially expressed genes ranked by the log2-transformed fold change between HRA and LRA samples revealed significantly enriched hallmarks of malignant tumors in HRA samples to include interferon gamma-mediated signaling pathway, inflammatory response, positive regulation of the JAK–STAT cascade, positive regulation of NIK/NF-kappa B signaling, and the tumor necrosis factor biosynthetic process. Meanwhile, functions, including carboxylic acid transport, detection of calcium ion, acid receptor signaling pathway, L-glutamate import and neuron cell–cell adhesion were related to the low RAS group (Figures 4C,D). Therefore, TARA infiltration might activate oncogenic pathways in glioma to promote malignant transformation.

### Characteristics of the immune micro-environment associated with TARA infiltration

**Figure 5:**
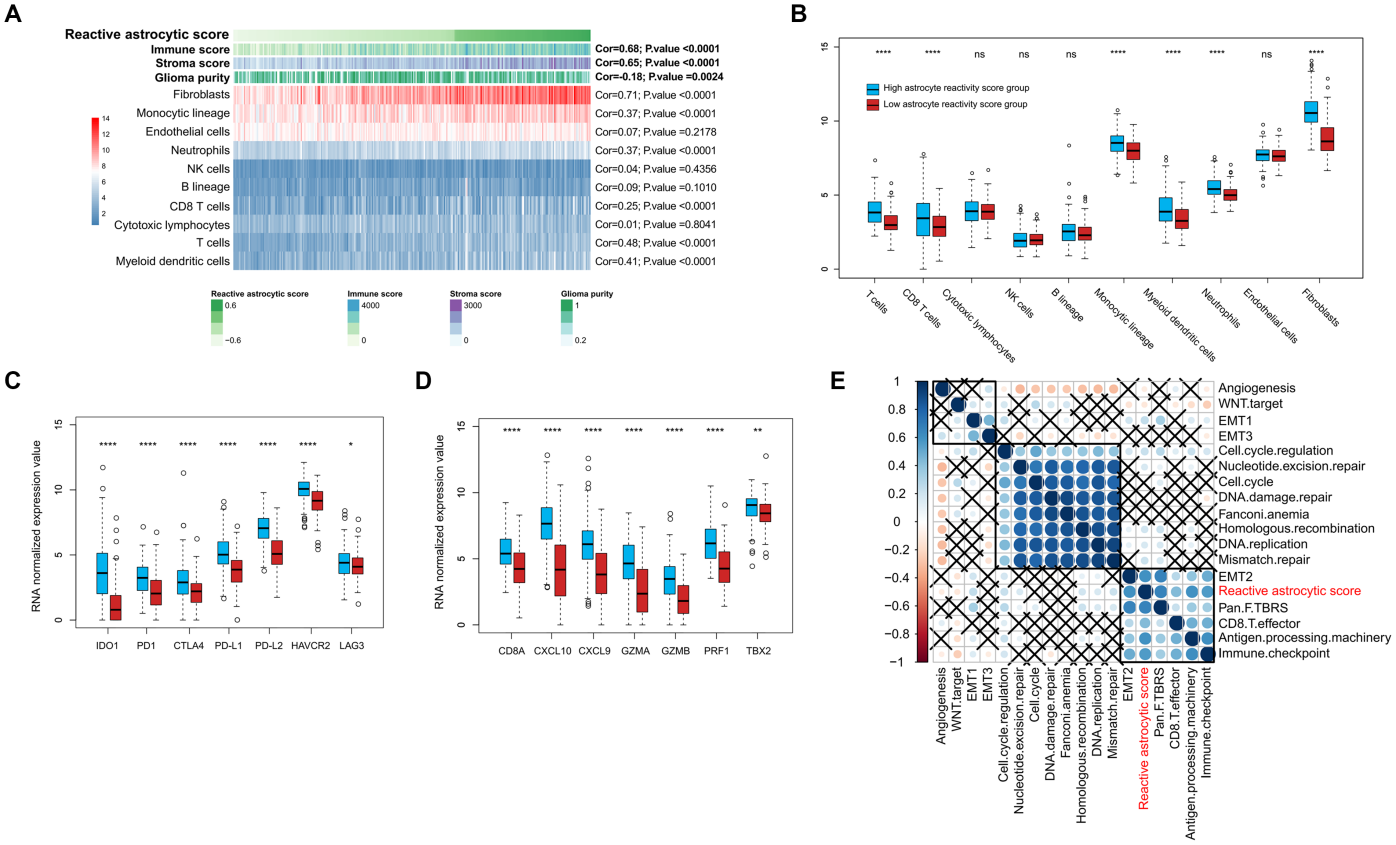
The tumor immune infiltrating characteristics of reactive astrocytic score. **(A)** The relationship between tumor microenvironment cells and reactive astrocytic score. **(B)** The distribution of immune cells between RAS high and low groups. **(C)** The immune-checkpoint relevant genes expressed in RAS high and low groups. **(D)** The immune activation relevant genes expressed in RAS high and low groups. **(E)** Correlations between RAS and gene signatures linked to epithelial-mesenchymal transition (EMT), immune checkpoint, mismatch repair, and immune activation. *p < 0.05; ****p < 0.0001; ns, not significant.

To determine the characteristics of the immune micro-environment associated with TARA infiltration, we deconvolved the immune cells with mRNA sequencing data using the MCPcounter method. The immune score (Cor = 0.68, *p* < 0.0001), stromal score (Cor = 0.65, *p* < 0.0001), fibroblasts (Cor = 0.71, *p* < 0.0001), monocytic lineage (Cor = 0.37, *p* < 0.0001), neutrophils (Cor = 0.37, *p* < 0.0001), CD8^+^ T cells (Cor = 0.68, *p* < 0.0001), T cells (Cor = 0.68, *p* < 0.0001) and myeloid dendritic cells (Cor = 0.68, *p* < 0.0001) all showed significantly positive correlation with reactive astrocyte score. Glioma purity showed significant negative correlation with the reactive astrocyte score (Figure 5A). Meanwhile, the HRA samples were characterized by increases in the infiltration of T cells, CD8^+^ T cells, monocytic lineage, myeloid dendritic cells, neutrophils, endothelial cells and fibroblasts (Figure 5B).

We then analyzed the differential expression of checkpoint relevant genes and cytokine and chemokine transcripts (22) between HRA and LRA groups (Figures 5C,D). HRA cases were associated with high expression levels of *IDO1*, *PD1*, *CTLA4*, *PD-L1*, *PD-L2*, *HAVCR2*, and *LAG3*. Moreover, the expression levels of cytokine and chemokine-related transcripts (*CD8A*, *CXCL10*, *CXCL9*, *GZMA*, *GZMB*, *PRF1*, and *TBX2*) were all significantly upregulated in the high RAS group. We also tested known signatures within TCGA dataset to better describe the functionality of the reactive astrocyte score. The results indicated that the reactive astrocyte score was significantly associated with mismatch repair, cell cycle regulation, epithelial-mesenchymal transition, CD8+ T effector, immune checkpoint, antigen processing machinery, and TGFb response signal (Figures 5A–E). These analyses indicated that patients with more reactive astrocyte infiltration are accompanied by immune infiltration and high activation of oncogenic signaling pathways.

### Increased TARA infiltration indicates poor prognosis and immune checkpoint inhibition

**Figure 6:**
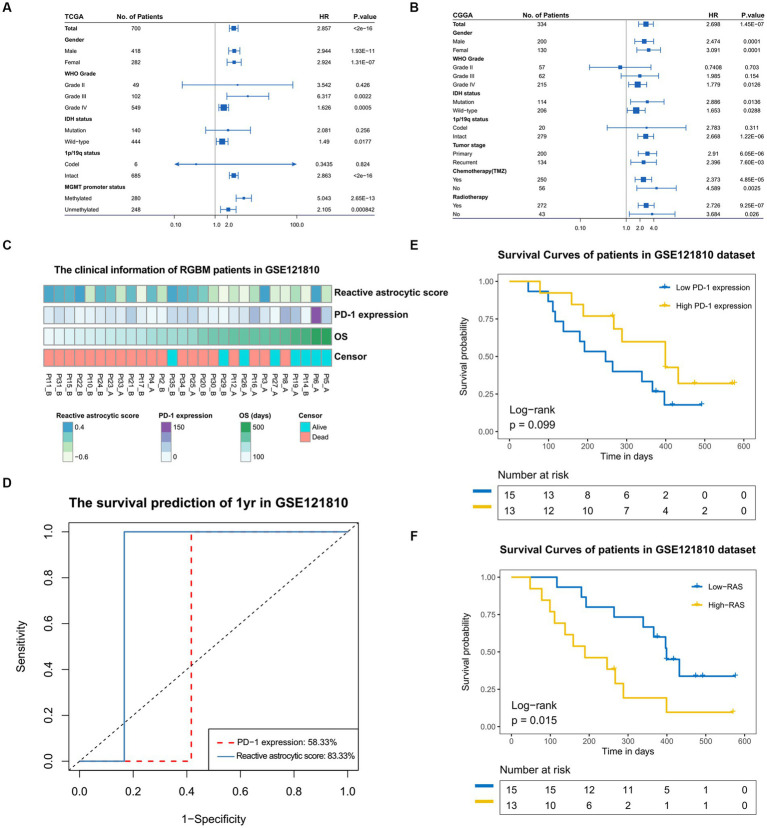
The prognostic value of reactive astrocytic score. **(A,B)** The forestplot exhibited the prognostic value of reactive astrocytic score in different clinical and molecular subgroups in TCGA and CGGA datasets. The length of the horizontal line represents the 95% confidence interval for each group. The vertical dotted line represents the hazard ratio (HR) of all patients. The vertical solid line represents HR = 1. HR > 1.0 indicate that high RAS is an unfavorable prognostic biomarker. Number of patients indicated. **(C)** The clinical information of RGBM patients in GSE121810 cohort. **(D)** The predictive value of the RAS and PD-1expression measured by ROC curves in the 121,810 cohort. **(E–F)** Kaplan-Meier survival analysis of PD-1 and RAS in GSE121810 cohort. Some patients were excluded from analysis for loss of follow-up.

We stratified glioma patients into distinct subgroups based on gender, WHO grades, *IDH* status, 1p/19q status, *MGMT* promotor status, tumor stage, chemotherapy and radiotherapy, and observed the prognostic value of the reactive astrocyte score as a continuous variable in TCGA and CGGA datasets (Figures 6A,B). The forest plot indicated that the reactive astrocyte score was a prognostic predictor in nearly all subgroups, except for WHO grade II and 1p/19q codeletion groups. Meanwhile, we used dichotomization to separate cases to depict the survival curves according to the median value and found that patients with higher reactive astrocyte scores had a significantly shorter overall survival than their counterparts in nearly all distinct subgroups in both datasets (Supplementary Figures S4, S5). The PD-1 immune checkpoint inhibitor can induce lasting tumor remission in patients with diverse advanced cancers (23). We therefore evaluated the predictive value of recurrent glioblastoma patients with neoadjuvant anti-PD-1 immunotherapy (GSE121810) (24). The clinical characteristics of recurrent glioblastoma patients are shown in Figure 6C. The time-dependent AUC indicated that the reactive astrocyte score had a high accuracy in predicting 1 year survival (Figure 6D). Furthermore, the reactive astrocyte score (Figure 6E) demonstrated a stronger power than *PD-1* expression (Figure 6F) in predicting recurrent glioblastoma patient overall survival with anti-PD-1 treatment. Patients with lower reactive astrocyte score were more likely to benefit from immune checkpoint treatment. Therefore, TARA infiltration might be a prognostic and a predictive marker for immune checkpoint inhibition.

## Discussion

Reactive astrocytes in neurological diseases are classified into two different types, termed A1 and A2, based on the activation status. A1 neuroinflammatory reactive astrocytes secrete chemokines and cytokines to promote tumorigenesis and metastasis, while neurotrophic factors upregulated in A2 reactive astrocytes promote survival and synapse repair (8, 25, 26). Recently, Heiland et al. (11) identified a distinct reactive astrocyte that is closely linked to JAK/STAT activation. These tumor-associated astrocytes can release a large number of anti-inflammation cytokines and promote an immunosuppressive environment in glioma.

In this study, we explored TARA infiltration in gliomas at both single-cell and bulk tumor levels using data from independent datasets. But technically, it was impossible to distinguish infiltrating TARA from neoplastic cells that are astrocytic-like, especially in bulk tumor sequencing data. We excluded cells with obvious copy number alterations (malignant cells). Single-cell RNA sequencing data showed a high proportion of TARA in the glioma micro-environment. Bulk tumor sample sequencing data showed that the high level of TARA infiltration was tightly associated with tumor malignancy factors, such as WHO IV grade, wild-type *IDH*, mesenchymal subtype, unmethylated *MGMT* and wild-type *ATRX*. Meanwhile, recurrent glioma patients maintained a higher level of TARAs than primary glioma patients. These phenomena indicated that neoplasm recurrence was closely related to a pro-inflammatory tumor environment. Analysis of genome instability indicated that patients with more TARA infiltration had more somatic mutation events and chromosome aberrations. The high amplification peak of *EGFR* (an oncogenic gene) and deletion peaks of *CDKN2A*, *CDKN2B*, and *PTEN* (tumor suppressor genes) were observed in HRA cases. TARA infiltration was a marker for global genomic instability in glioma. It has been suggested that molecularly distinct glioma subtypes exhibit differences in their microenvironment. Data from mouse models of GBM suggest that genetic driver mutations can create unique microenvironments (27). Genetic profiles, microenvironment, pathway activation were correlated in this study.

The Gene Ontology analysis of TARA infiltration showed that it was significantly associated with immune response, cytokine activation and infiltrating immune cell activities. In line with previous reports (11, 28, 29), the GSEA pathway analysis revealed that higher levels of TARA infiltration were accompanied with activation of the inflammatory response, the JAK–STAT cascade, NIK/NF-kappa B signaling and the tumor necrosis factor biosynthetic process. We elucidated a comprehensive landscape of interactions between TARAs and the tumor immune microenvironment. We found that more neutrophils, CD8+ cells and T cells were infiltrated in the HRA group. Meanwhile, cytokines that can recruit neutrophils, CD8+ cells and T cells, such as *CXCL9*, *CXCL10*, and *GZMA*, and checkpoints were significantly upregulated in the RAS high group. However, cytolytic lymphocytes that eliminate tumor cells were not upregulated in the HRA group. Neutrophils promote tumor growth and orchestrate resistance to anti-angiogenic therapies (30–32). TARAs had a tight association with glioma microenvironment activation. In addition, our study demonstrated that TARAs may influence glioma onset and recurrence via activation of neutrophils and dysfunction of cytolytic T cells. Therefore, blocking neutrophil infiltration may be advantageous in a combined treatment strategy.

Critically, our survival analysis revealed that patients with higher RAS had a significantly shorter overall survival than patients in the lower RAS group. We also observed that TARAs were a better predictive biomarker than PD-1, which will help advance precision immunotherapy for recurrent glioma patients. Immunological therapies that have shown promising results in other cancer types have turned out to be ineffective for glioma treatment. Our findings may help to facilitate the development of precision immunotherapy for glioma.

## Conclusion

In summary, our study comprehensively assessed TARAs in glioma, at both single cell and bulk tumor levels, by analyzing five independent datasets. The results showed TARAs to be tightly associated with glioma progression, immune therapy response and patient prognosis. Our findings will help guide the development of immunotherapy for glioma.

## Data availability statement

The original contributions presented in the study are included in the article/Supplementary material, further inquiries can be directed to the corresponding author.

## Ethics statement

Ethical review and approval was not required for the study on human participants in accordance with the local legislation and institutional requirements. Written informed consent from the patients/participants or patients/participants’ legal guardian/next of kin was not required to participate in this study in accordance with the national legislation and the institutional requirements.

## Author contributions

C-bZ, Z-lW, H-jL, ZW, and WJ contributed to the study conception and design. WJ designed and supervised the whole study. C-bZ and Z-lW did data downloading and analysis. H-jL and ZW participated in data interpretation. All authors contributed to the article and approved the submitted version.

## Funding

This study was supported by grants from the National Natural Science Foundation of China (No. 81802483, 81902528, 82071996) and Beijing Hospitals Authority Youth Program (No. QML20190507).

## Conflict of interest

The authors declare that the research was conducted in the absence of any commercial or financial relationships that could be construed as a potential conflict of interest.

## Publisher’s note

All claims expressed in this article are solely those of the authors and do not necessarily represent those of their affiliated organizations, or those of the publisher, the editors and the reviewers. Any product that may be evaluated in this article, or claim that may be made by its manufacturer, is not guaranteed or endorsed by the publisher.

## Footnotes

^1^http://www.cgga.org.cn

^2^https://tcga-data.nci.nih.gov/tcga/
