# Supplementary material for: Characterization of tumor-associated reactive astrocytes in gliomas by single-cell and bulk tumor sequencing
Source: Front Neurol. 2023 Jun 21;14:1193844. doi: 10.3389/fneur.2023.1193844 (PMC10320578; doi:10.3389/fneur.2023.1193844)
Supplement: Supplementary file 1 [file Data_Sheet_1.docx]

**Figure S1**.A, Total cell cluster in CGGA dataset; B, Cell type annotation by SingleR package.

**Figure S2**.The copy number analysis in CGGA datasets..

**Figure S3.** The clinical and molecular landscape of reactive astrocytic score in CGGA dataset. A, the samples in heatmap were arranged in order of increasing reactive astrocytic score. The relationships between reactive astrocytic score and patients’ features were evaluated (a, the distribution of reactive astrocytic score was assessed using the Student t test between two groups. b, the association between reactive astrocytic score and continuous variables was assessed using Pearson correlation tests. c, the distribution of glioma purity between several groups was assessed using one-way ANOVA). B-D, the distribution of reactive astrocytic score among different WHO grades, TCGA subtypes and molecular classifications. E-F, the distribution of reactive astrocytic score according PRS types. * indicated p< 0.05, ** indicated p< 0.01, ****, p< 0.0001.

**Figure S4.** Survival analysis of RAS in different subgroups in TCGA dataset, including who grades, male, female, WHO II grade, WHO III grade, WHO IV grade, IDH mutant, IDH wild-type, 1p/19q intact, MGMT methylated and MGMT unmethylated.

**Figure S5.** Survival analysis of RAS in different subgroups in CGGA dataset, including WHO grades, male, female, WHO grade II, WHO grade III, WHO grade IV, IDH mutant, IDH wild-type, 1p/19q intact, radiotherapy, without radiotherapy, chemotherapy, without chemotherapy, primary and recurrent tumor.
