# Supplementary figures and images for: Characterization of tumor-associated reactive astrocytes in gliomas by single-cell and bulk tumor sequencing
Source: Front Neurol. 2023 Jun 21;14:1193844. doi: 10.3389/fneur.2023.1193844 (PMC10320578; doi:10.3389/fneur.2023.1193844)

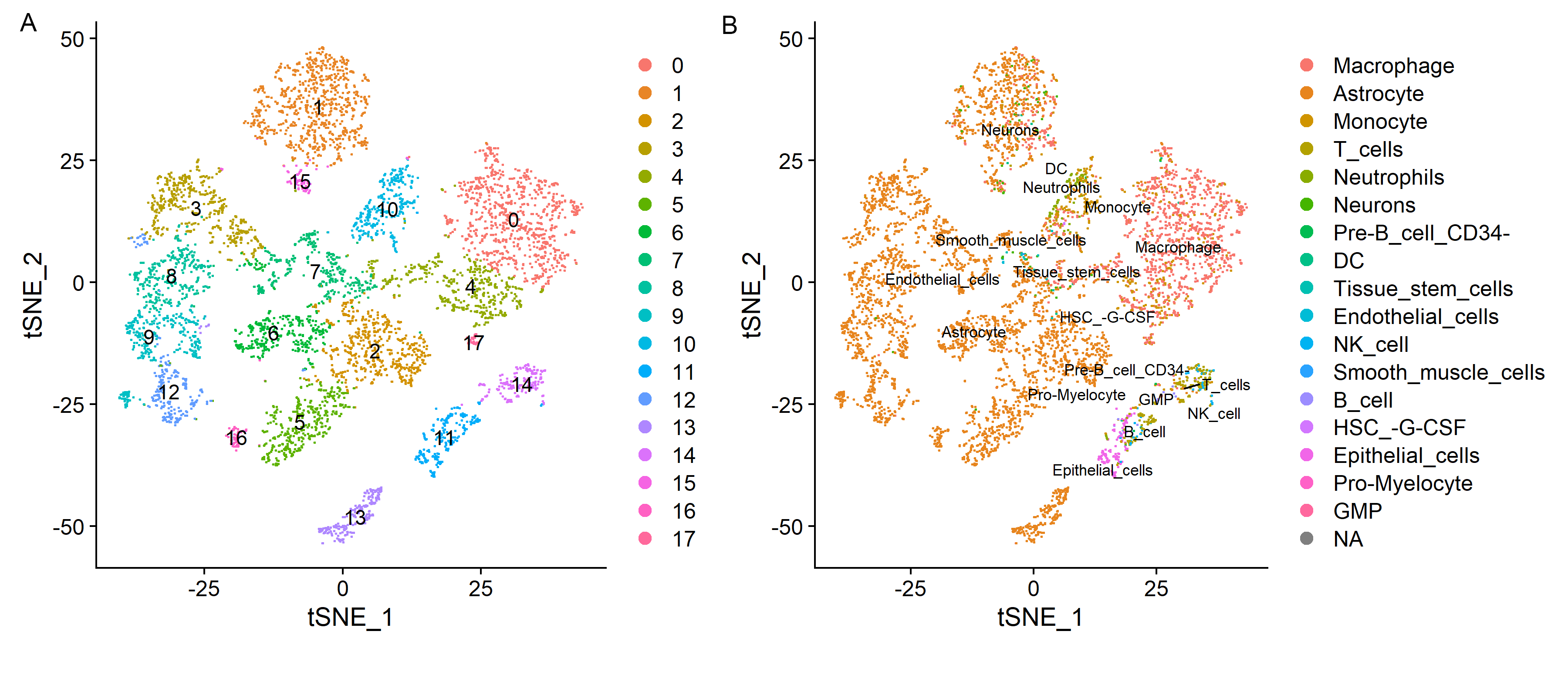

Supplement: Supplementary file 2 [file Image_1.TIF]

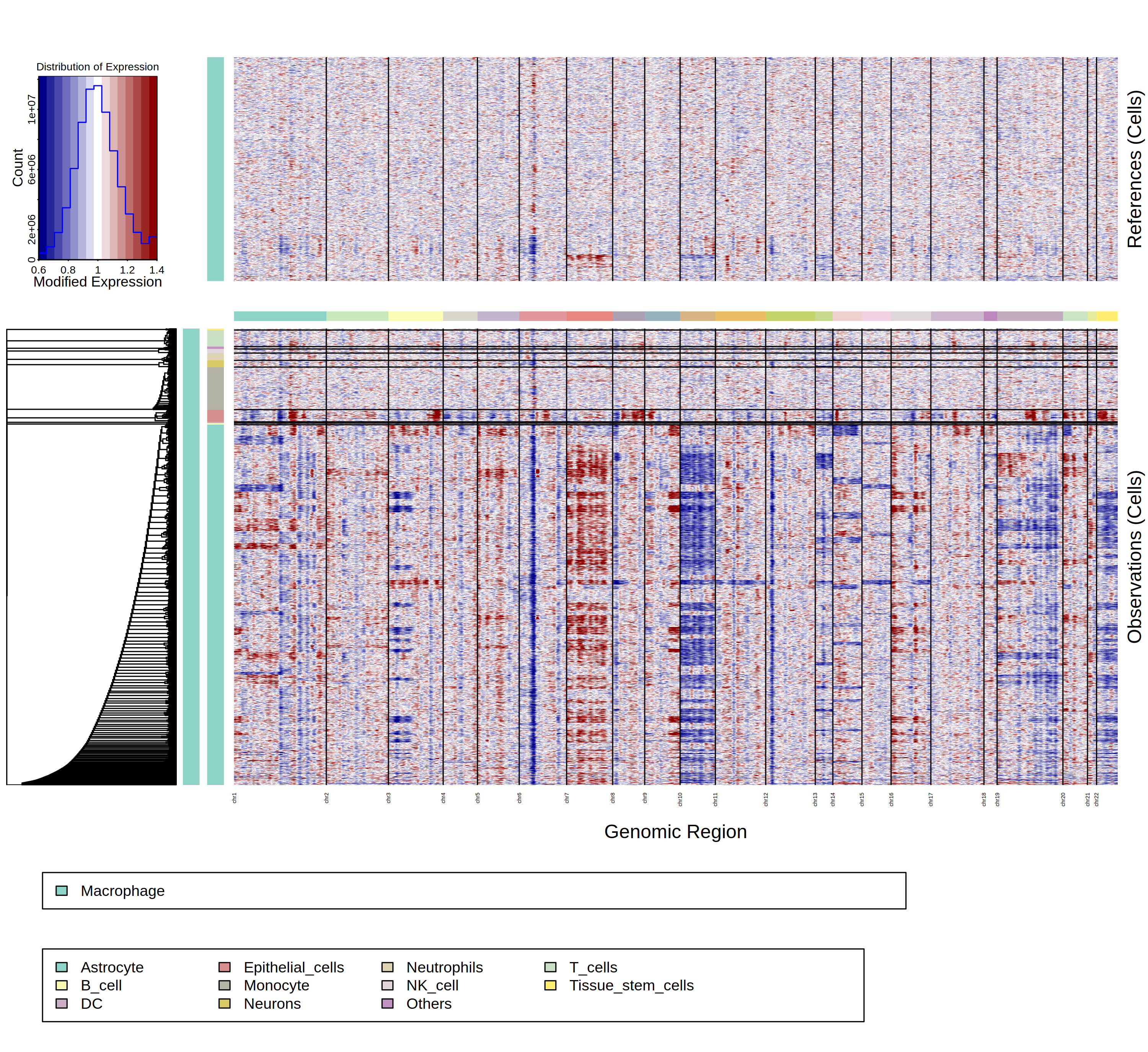

Supplement: Supplementary file 3 [file Image_2.TIFF]

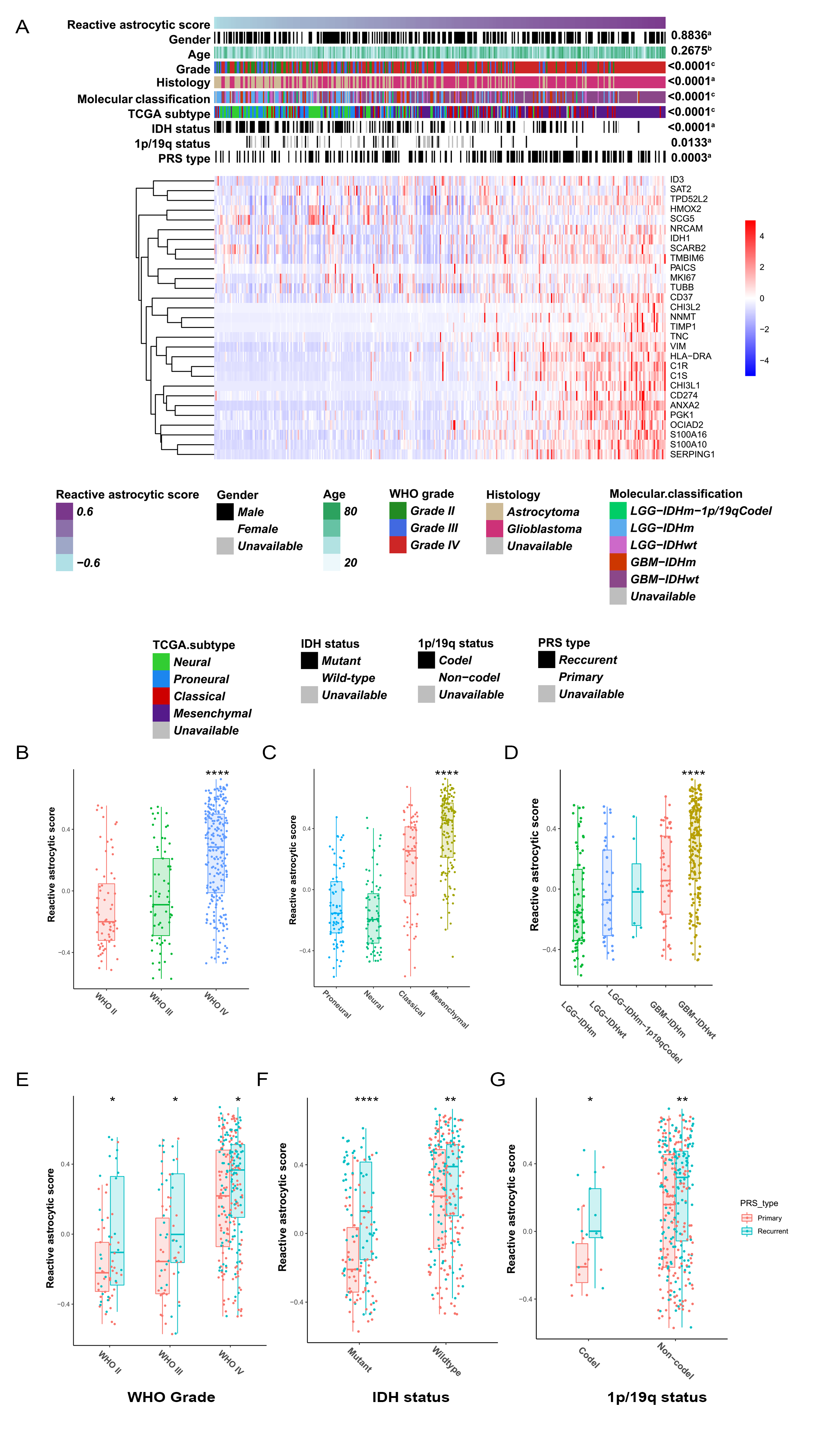

Supplement: Supplementary file 4 [file Image_3.TIF]

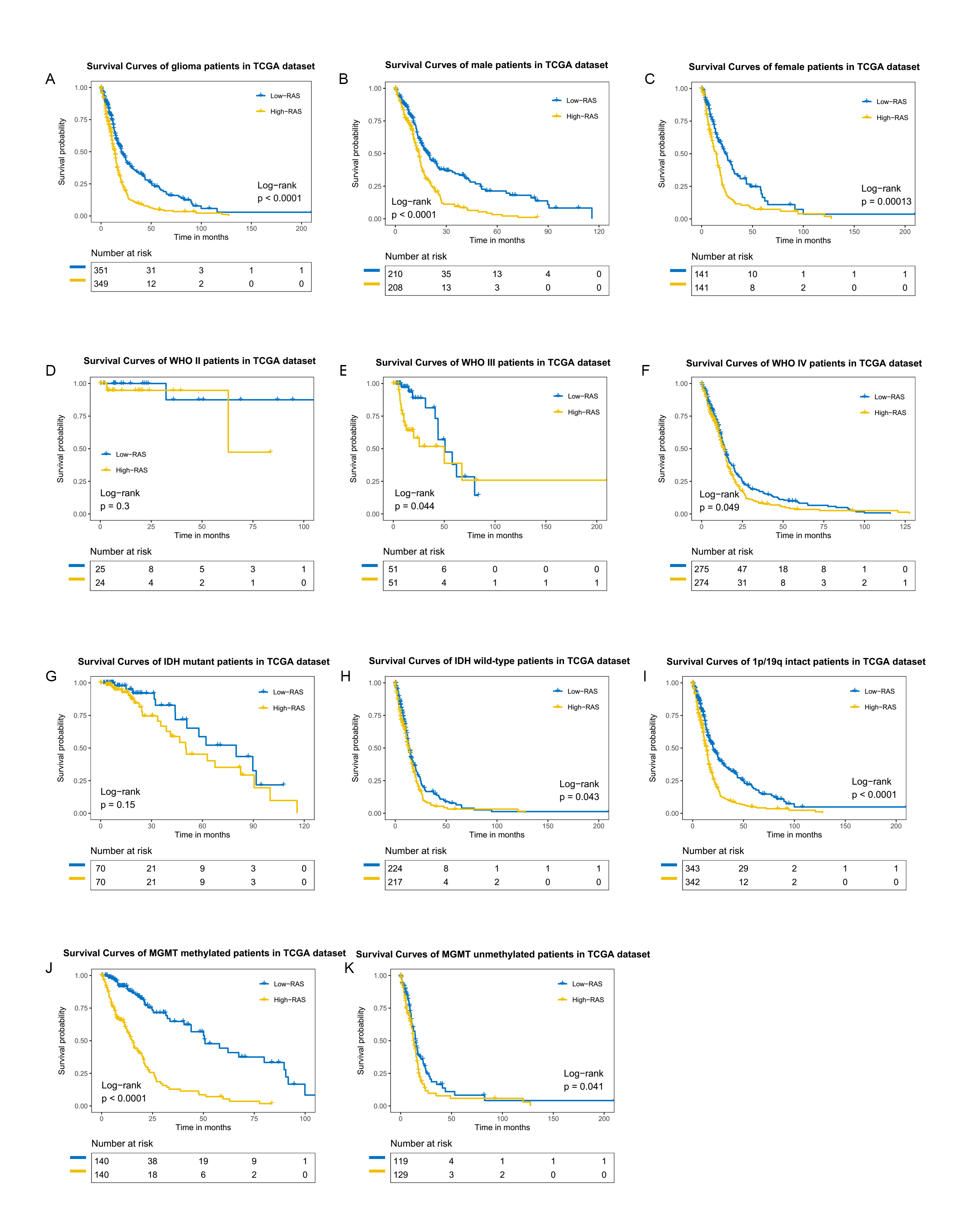

Supplement: Supplementary file 5 [file Image_4.TIF]

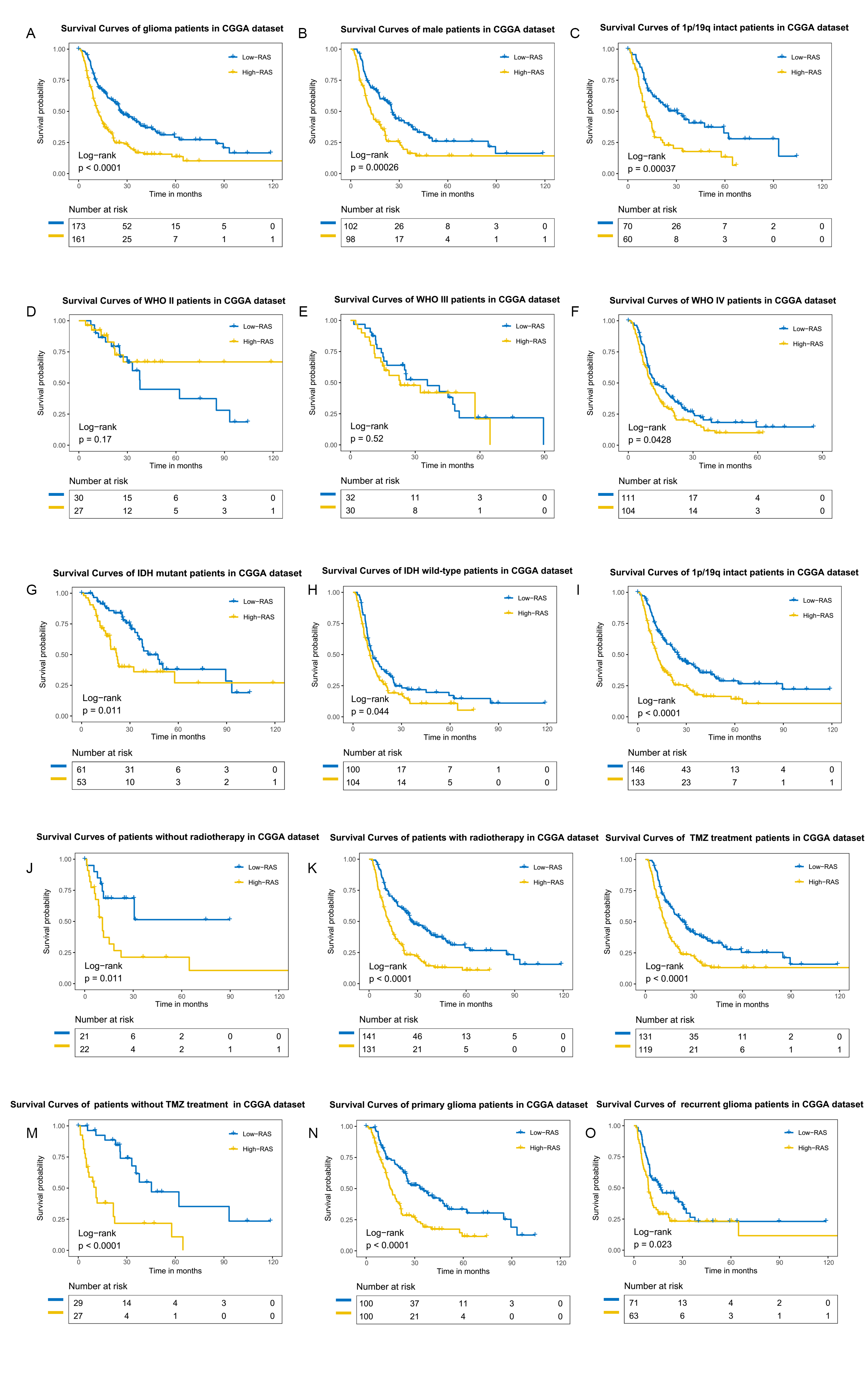

Supplement: Supplementary file 6 [file Image_5.TIF]
